# Using a Recurrent Neural Network To Inform the Use of Prostate-specific Antigen (PSA) and PSA Density for Dynamic Monitoring of the Risk of Prostate Cancer Progression on Active Surveillance

**DOI:** 10.1016/j.euros.2023.04.002

**Published:** 2023-04-29

**Authors:** Nikita Sushentsev, Luis Abrego, Anna Colarieti, Nimalan Sanmugalingam, Arnaldo Stanzione, Jeries Paolo Zawaideh, Iztok Caglic, Alexey Zaikin, Oleg Blyuss, Tristan Barrett

**Affiliations:** a Department of Radiology, Addenbrooke’s Hospital and University of Cambridge, Cambridge, UK; b Department of Women’s Cancer, Institute for Women’s Health, University College London, London, UK; c Unit of Radiology, IRCCS Policlinico San Donato, Milan, Italy; d Department of Advanced Biomedical Sciences, University of Naples Federico II, Naples, Italy; e Department of Radiology, IRCCS Ospedale Policlinico San Martino, Genoa, Italy; f Department of Mathematics, University College London, London, UK; g Wolfson Institute of Population Health, Queen Mary University of London, London, UK; h Department of Paediatrics and Paediatric Infectious Diseases, Sechenov First Moscow State Medical University, Moscow, Russia

**Keywords:** Prostate cancer, Active surveillance, Prostate-specific antigen, Predictive modelling, Longitudinal data, Artificial intelligence, Recurrent neural networks

## Abstract

The global uptake of prostate cancer (PCa) active surveillance (AS) is steadily increasing. While prostate-specific antigen density (PSAD) is an important baseline predictor of PCa progression on AS, there is a scarcity of recommendations on its use in follow-up. In particular, the best way of measuring PSAD is unclear. One approach would be to use the baseline gland volume (BGV) as a denominator in all calculations throughout AS (nonadaptive PSAD, PSAD_NA_), while another would be to remeasure gland volume at each new magnetic resonance imaging scan (adaptive PSAD, PSAD_A_). In addition, little is known about the predictive value of serial PSAD in comparison to PSA. We applied a long short-term memory recurrent neural network to an AS cohort of 332 patients and found that serial PSAD_NA_ significantly outperformed both PSAD_A_ and PSA for follow-up prediction of PCa progression because of its high sensitivity. Importantly, while PSAD_NA_ was superior in patients with smaller glands (BGV ≤55 ml), serial PSA was better in men with larger prostates of >55 ml.

**Patient summary:**

Repeat measurements of prostate-specific antigen (PSA) and PSA density (PSAD) are the mainstay of active surveillance in prostate cancer. Our study suggests that in patients with a prostate gland of 55 ml or smaller, PSAD measurements are a better predictor of tumour progression, whereas men with a larger gland may benefit more from PSA monitoring.

The past decade has witnessed a global increase in the uptake of active surveillance (AS) for management of patients with low-risk or favourable intermediate-risk prostate cancer (PCa) [1]. However, there is significant global variation in AS practices between centres and among guidelines [2], with the PRIAS study protocol most commonly used in Europe. Specifically, while some institutions favour protocol-driven biopsies to base their clinical decisions on histological ground truth, others argue for a more personalised approach in which the need for biopsy is guided by multiparametric magnetic resonance imaging (MRI) and prostate-specific antigen (PSA) kinetics [3]. While the latter strategy could indeed improve patient adherence to AS without compromising oncological outcomes, it requires the development of robust, dynamic, risk-adapted predictive models using high-quality multi-institutional data. Although highlighted as the current highest AS research priority [2], clinical translation of such models will require considerable time and resources. In parallel, application of longitudinal predictive modelling methods to existing MRI-driven AS cohorts can offer clinical insights that can shape future translational efforts.

We have encountered several clinical questions in our practice. First, while PSA density (PSAD) is an important baseline predictor of PCa progression on AS [4], [5], there is a scarcity of recommendations on its use during follow-up [2], and specifically on the best way of measuring MRI-derived PSAD. One approach would be to use baseline gland volume (BGV) as the denominator in all calculations throughout AS (nonadaptive PSAD, PSAD_NA_), while another would be to remeasure gland volume whenever a new MRI scan is performed (adaptive PSAD, PSAD_A_). Intuitively, PSAD_A_ is the preferred approach given its ability to provide more accurate values with dynamic increases in prostate volume in patients on AS [6]. However, PSAD_NA_ is easier to implement in routine clinical practice and there is no evidence regarding its comparative performance to either serial PSAD_A_ or PSA alone. In addition, the predictive performance of longitudinal PSA, PSAD_A_, or PSAD_NA_ may vary for different BGVs. Specifically, in patients with smaller prostates, even a modest increase in volume may lead to a considerable decrease in PSAD, while this effect would be the opposite in men with larger glands. In this study we tested these hypotheses using machine learning for longitudinal predictive modelling of the risk of PCa progression in patients on AS using serial PSA, PSAD_A_, and PSAD_NA_.

We included 332 patients enrolled on our previously described AS programme [4] between March 2012 and August 2020 in this ethically approved, single-centre study (Health Research Authority and Health and Care Research Wales, IRAS project ID 288,185; Supplementary Fig. 1). Clinical and histopathological characteristics of the study cohort are presented in Table 1. Over median follow-up of 51 mo (interquartile range 35–75) we collected 4508 serial PSA measurements (median 12 per patient) and performed 1362 serial prostate MRI scans (median 4 per patient). BGV for PSAD_NA_ and follow-up gland volumes for PSAD_A_ were calculated from MRI scans according to Prostate Imaging-Reporting and Data System guidelines [7] using three-plane measurements by four consultant urogenital radiologists with 4–14 yr of prostate MRI reporting experience. A previously described [8] long short-term memory recurrent neural network with leave-one-out cross-validation was applied to the data to generate areas under the receiver operating characteristic curve (AUCs) for predicting PCa progression on AS. Progression was noted in 80/332 patients, defined as either histopathological (biopsy-confirmed International Society of Urological Pathology grade group upgrading) or clear radiological stage progression (PRECISE [9] score of 5). Notably, repeat biopsies were performed at protocol-driven time points or were triggered earlier by a rise in PSA or suspected MRI progression [4]. AUCs were compared using DeLong’s test.

**Table 1:** Baseline clinicopathological characteristics of the study cohort a

Variable	Overall cohort (*n* = 332)	Progressors (*n* = 80)	Nonprogressors (*n* = 252)	*p* value
Median age, yr (IQR)	66 (61–69)	66 (62–69)	66 (61–69)	0.57
Median PSA, ng/ml (IQR)	5.6 (4.1–7.8)	5.8 (4.1–7.7)	5.5 (4.1–7.9)	0.59
Median BGV, ml (IQR)	45.8 (35.4–64.2)	42.0 (29.8–53.6)	49.6 (37.0–67.2)	0.007
Median PSAD, ng/ml/ml (IQR)	0.12 (0.08–0.17)	0.14 (0.09–0.22)	0.11 (0.08–0.16)	0.003
Median AS follow-up, mo (IQR)	51.0 (35.0–75.8)	43.5 (28.5–59.0)	56.5 (38.0–79.8)	0.0002
Biopsy ISUP grade 1, *n* (%)	220 (66)	48 (60)	172 (68)	0.18
Biopsy ISUP grade 2, n (%)	112 (34)	32 (40)	80 (32)	

BGV = baseline gland volume; IQR = interquartile range; ISUP = International Society of Urological Pathology; PSA = prostate-specific antigen; PSAD = PSA density.

a Intergroup comparisons of patient characteristics were performed using the Mann-Whitney U test and Fisher’s exact test, as appropriate.

Prostate volume increased over time (Fig. 1A), consistent with previous results [6]. At the cohort level, serial PSAD_NA_ significantly outperformed both PSAD_A_ and PSA for prediction of PCa progression (*p* < 0.0001 for all; Fig. 1A,B and Supplementary Table 1). To assess the impact of BGV on biomarker performance, we a priori defined three BGV cutoffs (group A, ≤40 ml; group B, 41–55 ml; group C, >55 ml) to divide the cohort into three groups of similar sample size and distribution of progressors and nonprogressors (Supplementary Table 2). In groups A and B, PSAD_NA_ showed significantly better performance in comparison to both PSAD_A_ and PSA (*p* < 0.0001 for all; Fig. 1C and Supplementary Table 3). This can be explained by the higher sensitivity of PSAD_NA_ (Fig. 1B,C), which effectively overestimates the “true” PSAD by maximising the impact of increasing PSA with a stable denominator of BGV. Conversely, in group C, PSA significantly outperformed both PSAD_A_ and PSAD_NA_ (*p* < 0.0001 for all; Fig. 1C and Supplementary Table 3). This probably reflects the need for a much higher relative increase in PSA to change PSAD values sufficiently to match the more rapidly increasing gland volume in patients with BGV >55 ml (Supplementary Fig. 2). Importantly, the diagnostically superior PSAD_NA_ and PSA are easier to use clinically given the inconsistent reporting of follow-up gland volumes as required for calculating PSAD_A_.

**Fig. 1:**
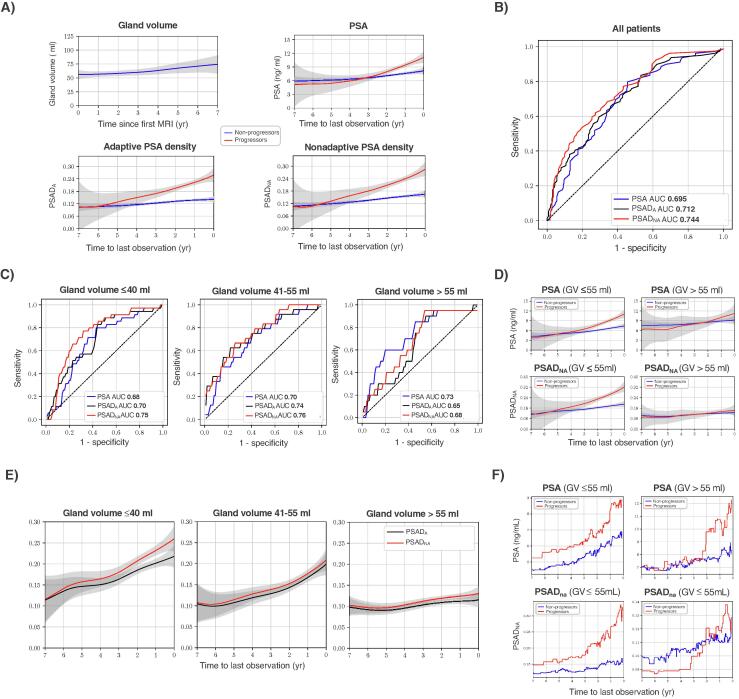
Use of serial PSA and PSAD for predicting prostate cancer progression on active surveillance. (A) LOWESS curves demonstrating serial changes in prostate GV, PSA, PSAD_A_, and PSAD_NA_ for patients with and without progression. (B) ROC curves for serial PSA, PSAD_A_, and PSAD_NA_ applied to the whole cohort to assess the ability to predict prostate cancer progression in patients on active surveillance. (C) ROC curves for serial PSA, PSAD_A_, and PSAD_NA_ for patients with differing BGV. (D) LOWESS curves demonstrating changes in serial PSA and PSAD_NA_ for patients with smaller (≤55 ml) and larger (>55 ml) BGV. (E) LOWESS curves demonstrating the difference between PSAD_A_ and PSAD_NA_ by BGV. (F) Serial changes in median PSA and PSAD_NA_ for patients with smaller (≤55 ml) and larger (>55 ml) BGV. AUC = area under the ROC curve; BGV = baseline GV; GV = gland volume; LOWESS = locally weighted scatterplot smoothing; PSA = prostate-specific antigen; PSAD = PSA density (in ng/ml/ml); PSAD_A_ = adaptive PSAD; PSAD_NA_ = nonadaptive PSAD; ROC = receiver operating characteristic.

These findings can be visualised in locally weighted scatterplot smoothing curves that show more prominent differences in longitudinal trends for PSA and PSAD_NA_ between progressors and nonprogressors in patients with smaller (≤55 ml) and larger (>55 ml) BGV (Fig. 1D). The same trend is evident from plots of dynamic changes in median PSA and PSAD_NA_ values (Fig. 1F). For patients with smaller glands, PSAD_NA_ grew steadily in progressors and plateaued in nonprogressors, while PSA showed a proportionate increase in both groups until the last year before progression/censorship. This trend was reversed for patients with larger glands: median PSA showed a much clearer relative increase in progressors in comparison to PSAD_NA_. Interestingly, in group A the difference between serial PSAD_NA_ and PSAD_A_ was considerably larger than in groups B and C, for which the two methods produced similar values (Fig. 1E).

Our study has several limitations, including its single-centre nature, retrospective design, limited sample size, and lack of assessment of inter-reader variability for MRI-derived gland volume measurement (which is generally >0.90 for expert readers [10]). While identifying specific serial PSAD_NA_ and PSA cutoffs sufficient to trigger unscheduled MRI or biopsy was beyond the scope of this study, our data provide several observations to be tested in future work. First, regardless of BGV, a consistent increase in PSAD_NA_ beyond the median value of 0.18 ng/ml/ml was a characteristic feature of progressors that could be first noted 3 yr before their clinical reclassification (Fig. 1A,D,F). Second, albeit less pronounced, a similar trend was observed for the median PSA value of 9 ng/ml; this was breached approximately 2 yr before clinical progression in patients with BGV of >55 ml (Fig. 1F).

Overall, this study offers three main observations:

• Dynamic monitoring of PSAD_NA_ consistently outperformed PSAD_A_ in predicting PCa progression on AS both for the whole AS population and in particular for patients with BGV ≤55 ml.
• Patients with BGV >55 ml benefit more from serial PSA monitoring, since PSAD is more stable and less predictive as the volume has a higher denominator value.
• If clinicians prefer more accurate PSAD, they should prioritise measurement of PSAD_A_ in patients with BGV of ≤40 ml, for whom the discrepancy with PSAD_NA_ is more pronounced.

These results may help in informing both current clinical practice and future multicentre studies to develop personalised AS algorithms using dynamic risk-adapted predictive modelling and incorporating all available clinical data, including serial MRI and biopsy results.

  ***Author contributions***: Nikita Sushentsev had full access to all the data in the study and takes responsibility for the integrity of the data and the accuracy of the data analysis.

  *Study concept and design*: Sushentsev, Zaikin, Blyuss, Barrett.

*Acquisition of data*: Sushentsev, Colarieti, Sanmugalingam, Stanzione, Zawaideh, Caglic.

*Analysis and interpretation of data*: Sushentsev, Abrego, Caglic, Zaikin, Blyuss, Barrett.

*Drafting of the manuscript*: Sushentsev.

*Critical revision of the manuscript for important intellectual content*: Abrego, Caglic, Zaikin, Blyuss, Barrett.

*Statistical analysis*: Abrego, Zaikin, Blyuss.

*Obtaining funding*: Sushentsev, Zaikin, Blyuss, Barrett.

*Administrative, technical, or material support*: Zaikin, Blyuss, Barrett

*Supervision*: Zaikin, Blyuss, Barrett.

*Other*: None.

  ***Financial disclosures:*** Nikita Sushentsev certifies that all conflicts of interest, including specific financial interests and relationships and affiliations relevant to the subject matter or materials discussed in the manuscript (eg, employment/affiliation, grants or funding, consultancies, honoraria, stock ownership or options, expert testimony, royalties, or patents filed, received, or pending), are the following: None.

  ***Funding/Support and role of the sponsor*:** This research was supported by the National Institute for Health and Care Research (NIHR) Cambridge Biomedical Research Centre (NIHR203312). The study sponsor did not play a role in data collection and analysis. The views expressed are those of the authors and not necessarily those of the NIHR or the Department of Health and Social Care. The authors also acknowledge support from Cancer Research UK (CRUK; Cambridge Imaging Centre grant number C197/A16465; ACED Pilot Award A095792/EICEDAAP\100009), the CRUK National Cancer Imaging Translational Accelerator (C42780/A27066), and the Engineering and Physical Sciences Research Council Imaging Centre in Cambridge and Manchester. Nikita Sushentsev acknowledges support from the Gates Cambridge Trust. Oleg Blyuss acknowledges support from Cancer Research UK and EPSRC joint award EDDCPJT/100022.
