# Supplementary material for: Using a Recurrent Neural Network To Inform the Use of Prostate-specific Antigen (PSA) and PSA Density for Dynamic Monitoring of the Risk of Prostate Cancer Progression on Active Surveillance
Source: Eur Urol Open Sci. 2023 Apr 29;52:36–9. doi: 10.1016/j.euros.2023.04.002 (PMC10172696; doi:10.1016/j.euros.2023.04.002)
Supplement: Supplementary data 1 [file mmc1.docx]

**Supplementary material**

**Supplementary Fig. 1.** Study flowchart.

AS = active surveillance; ISUP = International Society of Urological Pathology; MRI = magnetic resonance imaging; PSA = prostate-specific antigen.

**Supplementary Fig. 2.** Locally weighted scatterplot smoothing curves of the serial change in prostate volume for patients with baseline gland volume (BGV) ≤55 ml (blue) and >55 ml (red).

**Supplementary Table 1 –**AUC for PSA, PSAD_A_, and PSAD_NA_ for prediction of prostate cancer progression in active surveillance at the whole cohort level

| **Model** | **AUC (95% CI)** | **Comparator** | ***p* value (DeLong’s test)** |
| --- | --- | --- | --- |
| PSA | 0.695 (0.630–0.758) | PSAD_A_ | <0.0001 |
| PSAD_A_ | 0.712 (0.647–0.775) | PSAD_NA_ | <0.0001 |
| PSAD_NA_ | 0.745 (0.683–0.804) | PSA | <0.0001 |

AUC = area under the receiver operating characteristic curve; CI = confidence interval; PSA = prostate-specific antigen; PSAD = PSA density; PSAD_A_ = adaptive PSAD; PSAD_NA_ = nonadaptive PSAD.

**Supplementary Table 2.** Baseline clinicopathological characteristics of the three patient groups divided by the baseline gland volume

| **Variable** | **BGV ≤40 ml** | **BGV 41–55 ml** | **BGV >55 ml** |
| --- | --- | --- | --- |
| Patients, *n* (% of whole cohort) | 122 (37) | 92 (28) | 118 (35) |
| Progressors, *n* (% of the group) | 35 (29) | 25 (27) | 20 (17) |
| Nonprogressors, *n* (% of the group) | 87 (71) | 67 (73) | 98 (83) |
| Median age, yr (IQR) | 63 (58–66) | 65 (61–67) | 66 (62–70) |
| Median PSA, ng/ml (IQR) | 4.3 (3.3–6.2) | 5.1 (4.2–7.4) | 7.0 (5.2–9.6) |
| Median gland volume, ml (IQR) | 31.0 (24.0–36.5) | 46.3 (43.1–50.1) | 71.8 (63.4–88.1) |
| Median PSAD, ng/ml/ml (IQR) | 0.16 (0.10–0.21) | 0.11 (0.09–0.15) | 0.09 (0.07–0.14) |
| Median AS follow-up, mo (IQR) | 50.0 (34.8–69.3) | 50.0 (35.0–73.0) | 59.0 (35.8–84.0) |
| Biopsy ISUP grade 1, *n* (% of group) | 79 (65) | 56 (61) | 85 (72) |
| Biopsy ISUP grade 2, *n* (% of group) | 43 (35) | 36 (39) | 33 (28) |

AS = active surveillance; BGV = baseline gland volume; IQR = interquartile range; ISUP = International Society of Urological Pathology; PSA = prostate-specific antigen; PSAD = PSA density.

**Supplementary Table 3 –** AUCs for PSA, PSAD_A,_ and PSAD_NA_ by BGV for predicting prostate cancer progression in active surveillance

| BGV | Model | AUC | Comparator | *p* value (DeLong’s test) |
| --- | --- | --- | --- | --- |
| ≤40 ml | PSA | 0.675 | PSAD_A_ | <0.0001 |
|  | PSAD_A_ | 0.7 | PSAD_NA_ | <0.0001 |
|  | PSAD_NA_ | 0.753 | PSA | <0.0001 |
| 41–55 ml | PSA | 0.7 | PSAD_A_ | <0.0001 |
|  | PSAD_A_ | 0.741 | PSAD_NA_ | <0.0001 |
|  | PSAD_NA_ | 0.758 | PSA | <0.0001 |
| >55 ml | PSA | 0.731 | PSAD_A_ | <0.0001 |
|  | PSAD_A_ | 0.648 | PSAD_NA_ | <0.0001 |
|  | PSAD_NA_ | 0.675 | PSA | <0.0001 |

AUC = area under the receiver operating characteristic curve; BGV = baseline gland volume; PSA = prostate-specific antigen; PSAD = PSA density; PSAD_A_ = adaptive PSA; PSAD_NA_ = nonadaptive PSAD.
